# Nanoscale Restructuring of Polymer Materials to Produce Single Polymer Composites and Miscible Blends

**DOI:** 10.3390/biom9060240

**Published:** 2019-06-19

**Authors:** Alan E. Tonelli

**Affiliations:** Fiber & Polymer Chemistry Program, Wilson College of Textiles, North Carolina State University, Raleigh, NC 27606-8301, USA; atonelli@ncsu.edu

**Keywords:** polymer inclusion compounds, coalesced polymers, self-nucleation, miscible blending, single polymer composites

## Abstract

I summarize work conducted in our laboratories over the past 30 years using small host molecules to restructure polymer materials at the nanometer level. Certain small molecules, such as the cyclic starches cyclodextrins (CDs) and urea (U) can form non-covalent crystalline inclusion compounds (ICs) with a range of guest molecules, including many polymers. In polymer-CD- and -U-ICs, guest polymer chains reside in narrow channels created by the host molecule crystals, where they are separated and highly extended. When the host crystalline lattice is carefully removed, the guest polymer chains coalesce into a bulk sample with an organization that is distinct from that normally produced from its melt or from solution. Amorphous regions of such coalesced polymer samples have a greater density, likely with less chain entanglement and more chain alignment. As a consequence, after cooling from their melts, coalesced amorphous polymers show glass-transition temperatures (T_g_s) that are elevated above those of samples prepared from their solutions or melts. Upon cooling from their melts, coalesced samples of crystallizable polymers show dramatically-increased abilities to crystallize more rapidly and much closer to their melting temperatures (T_m_s). These unique behaviors of polymers coalesced from their CD- and U-ICs are unexpectedly resistant to extended annealing above their T_g_s and T_m_s. Taking advantage of this behavior permits us to create polymer materials with unique and improved properties. Among these are amorphous polymers with elevated T_g_s and semi-crystalline polymers with finer more uniform morphologies. Improved mechanical properties can be achieved through self-nucleation with small amounts of the same polymer made rapidly crystallizable through coalescence from its CD- or U-IC. This can lead to single polymer composites with as-received polymer matrices and self-nucleated reinforcements. Through simultaneous formation and subsequent coalescence from their common CD–ICs, stable well-mixed blends can be achieved between any two or more polymers, despite their inherent immiscibilities. Such coalesced and well-mixed blends are also resistant to phase segregation when heated for extensive periods well above their T_g_s and T_m_s.

## 1. Introduction

For nearly 30 years, I have been interested in the non-covalently bonded inclusion compounds (ICs) formed between small host molecules and, in particular, their ICs formed with polymer guests [1]. These polymer-ICs are not held together by chemical bonds [2]. Rather, they are believed to be the result of weaker forces that are essentially steric in origin, i.e., related not just to the chemical natures, but also to the shapes and dimensions of the guest molecules [3].

The stability of ICs is thought to depend on competing van der Waals interactions between the host and guest and the force of internal and external hydrogen bonds among and between the host(s). Because the van der Waals energy varies as the inverse sixth power of the distance, a necessary tight fit between the host and guest molecules is indicated. Hence, the stability within the cavity of the host molecule, or formed from the host crystalline lattice, is directly related to the sizes and the shapes of the guests [4]. 

**Figure 1:**
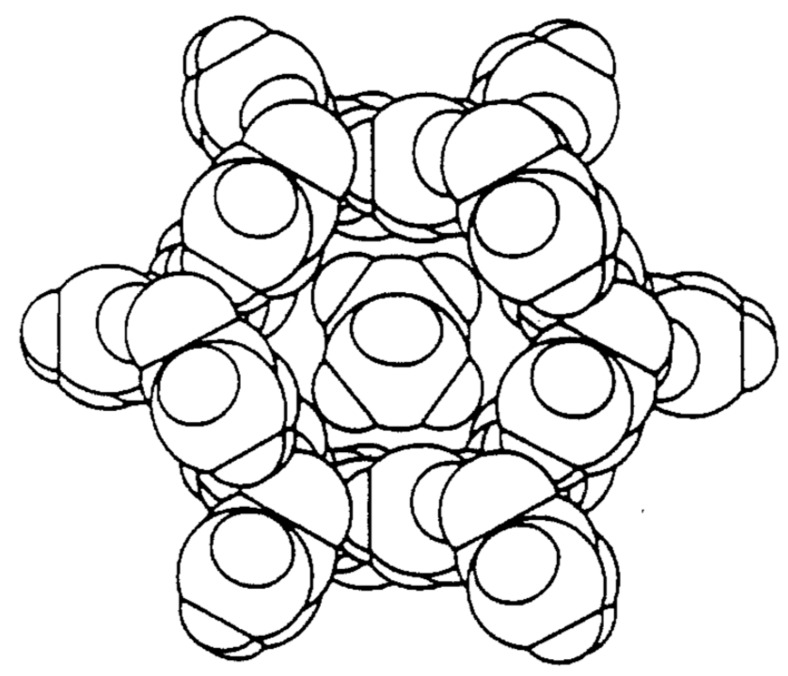
Space-filling drawing of a channel in the urea-n-hexadecane-inclusion compound (IC). Reproduced with permission from [14]. Copyright 1989 Elsevier.

**Figure 2:**
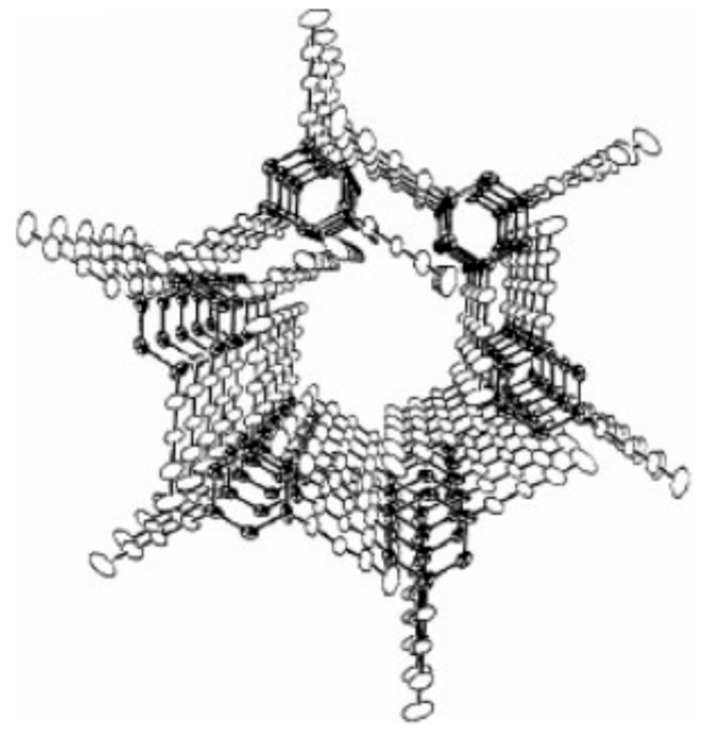
Schematic perspective view down one channel that penetrates the hexagonal crystalline lattice of tris(o-phenylenedioxy)cyclotriphos-phazene (TPP). Reproduced with permission from [10]. Copyright 1985 American Chemical Society.

**Figure 3:**
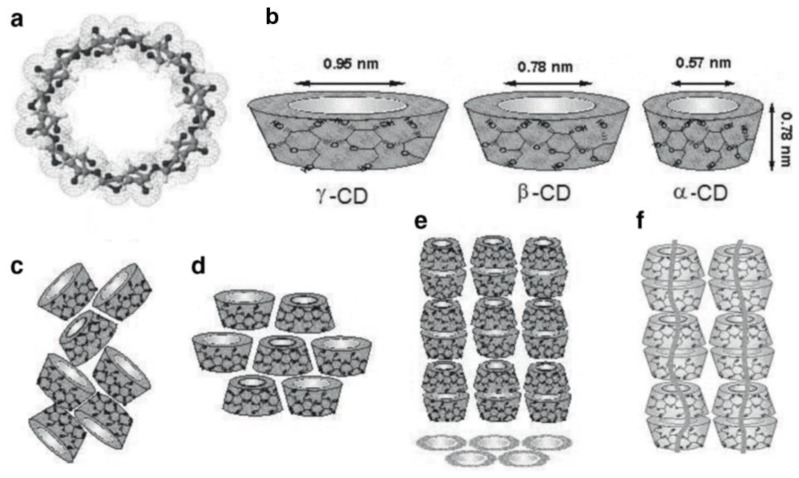
(**a**) γ-cyclodextrin (CD) chemical structure and (**b**) approximate dimensions of α-, β-, and γ-CDs; schematic representation of packing structures of (**c**) cage-type, (**d**) layer-type, and (**e**) head-to-tail channel type CD crystals; and (**f**) CD–IC channels containing included polymer guests. Reproduced with permission from [15]. Copyright 2009 Springer.

Most widely known among IC hosts are urea (U) [5,6], thiourea (TU) [5,6], perhydrotriphenylene (PHTP) [7,8,9], the cyclo-triphosphazines (TPP) [10], and cyclodextrins (CDs) [1,2], all of which form narrow crystalline channels around their nearly fully extended guest polymer chains (see Figure 1, Figure 2 and Figure 3). Though some polymers with narrow cross-sections may form ICs with host γ-CD that contain two side-by-side chains in each channel [11,12,13], in most polymer-CD–ICs, single stretched and isolated chains are included in each crystalline host channel.

It should be noted that neat CDs contain a small number of water molecules in their largely hydrophobic cavities [16,17]. Replacement of this cavity water by more hydrophobic guests, including polymers, likely also contributes to the formation and stability of CD–ICs [3]. 

**Figure 4:**
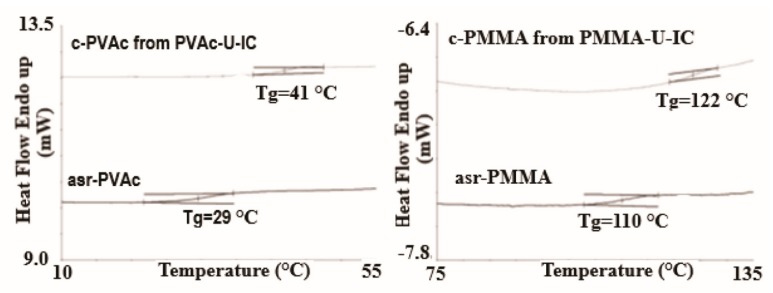
DSC observed glass transitions in amorphous poly (vinyl acetate)(PVAc) and Poly (Methyl methacrylate) (PMMA) as-received (asr) and coalesced (c) from their urea (U)-ICs. Reproduced with permission from [23]. Copyright 2013 Wiley Periodicals, Inc.

**Figure 5:**
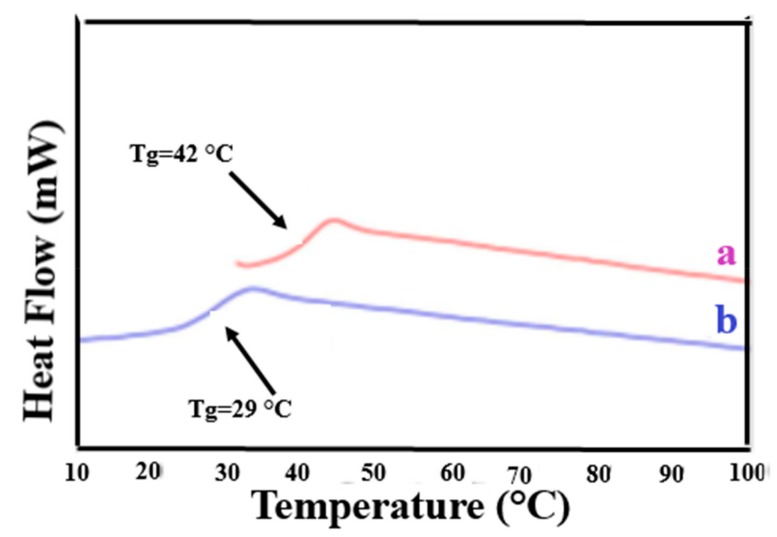
DSC thermograms of the second heating scan of: (**a**) c-PVAc from its γ-CD–IC; and (**b**) asr-PVAc. Reproduced with permission from the publisher [24]. Copyright 2005 Elsevier Ltd.

**Figure 6:**
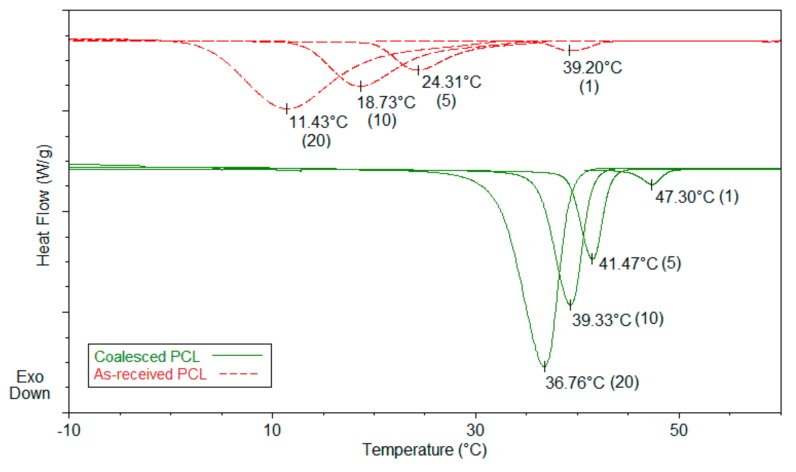
Melt-crystallization curves of as-received and coalesced poly(ε-caprolactone) (PCL) observed at 20, 10, 5, and 1 °C/min cooling rates. Reproduced with permission from [25]. Copyright 2011 Elsevier Ltd.

Polymer chains included in their crystalline ICs, in addition to having conformations and mobilities distinct from their bulk samples [18], may, by the careful removal of the host crystalline lattice, be coalesced into bulk samples, which also behave distinctly from bulk samples obtained from their solutions and melts [15,19,20,21,22]. Such coalesced amorphous and semi-crystalline polymers exhibit elevated glass-transion temperatures (T_g_s) (see Figure 4 and Figure 5) and enhanced crystallizabilities (see Figure 6), respectively. 

Initially surprising were the observations that the distinct behaviors of bulk polymer samples made by coalescence from their ICs were resistant to long-time annealing at temperatures above their T_g_s and melting temperature (T_m_s) [15,20,21,22].

For example, the disparity between the T_g_s of as-received and coalesced samples of PVAc and poly(methyl methacrylate (PMMA) displayed in Figure 4 and Figure 5, remained after weeks of annealing well above their T_g_s [23,24]. The more rapid crystallization of coalesced poly(ε-caprolactone) (PCL) seen in Figure 6 was also not affected by long duration melt annealing of coalesced PCL at 100 °C [25].

**Figure 7:**
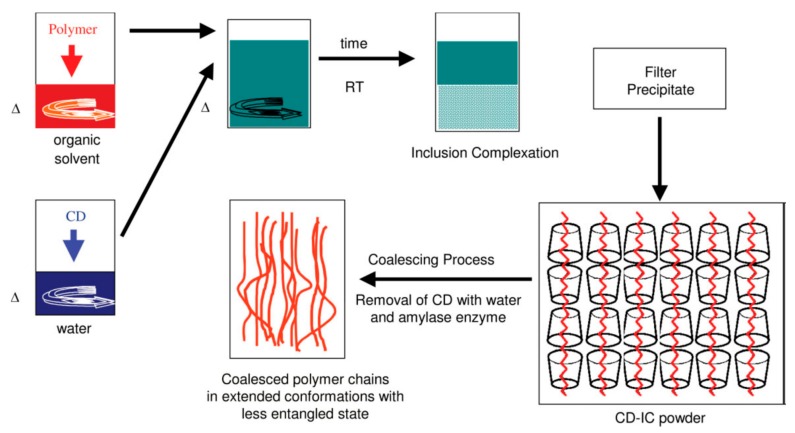
Schematic representation of polymer-CD IC formation, the coalescence process, and the bulk coalesced polymer. Reproduced with permission from [27]. Copyright 2005 Elsevier Ltd.

A reason was presented for the resistance to high temperature annealing of polymers coalesced from their CD–ICs and depicted in Figure 7 [21,26]. It was suggested that, though the initially coalesced, largely extended, separated, and un-entangled chains likely random-coil relatively rapidly, the center-of-mass diffusion that must accompany full entanglement of their chains is extremely sluggish. This was concluded to be the source of the subsequent slow establishment of homogeneous well-mixed melts from coalesced samples consisting of small randomly arranged regions containing somewhat aligned un-entangled chains. The process of entangling the largely separated and not fully interpenetrating randomly coiled chains coalesced from their CD–ICs is apparently particularly slow: much slower in fact than the center-of mass diffusion of polymer chains in their fully entangled melts.

Regardless of the validity of the above suggestion [21,26], in the remainder of this review of the behavior and uses of polymers restructured via coalescence from their crystalline ICs, the focus will be on the creation of well-mixed polymer blends and the production of single polymer composites. The former produced by first forming and then coalescing two or more polymers from their common ICs, and the latter by using rapidly crystallizable coalesced polymers, such as PCL shown in Figure 6, as self-nucleants to create rapidly crystallizable polymer samples with finer more homogeneous semi-crystalline morphologies with improved mechanical properties. 

## 2. Materials and Methods 

Experimental details, including materials purchased and synthesized and the means used in their characterization, are provided in the references.

## 3. Results and Discussion

### 3.1. Compatible Coalesced Polymer Blends

**Figure 8:**
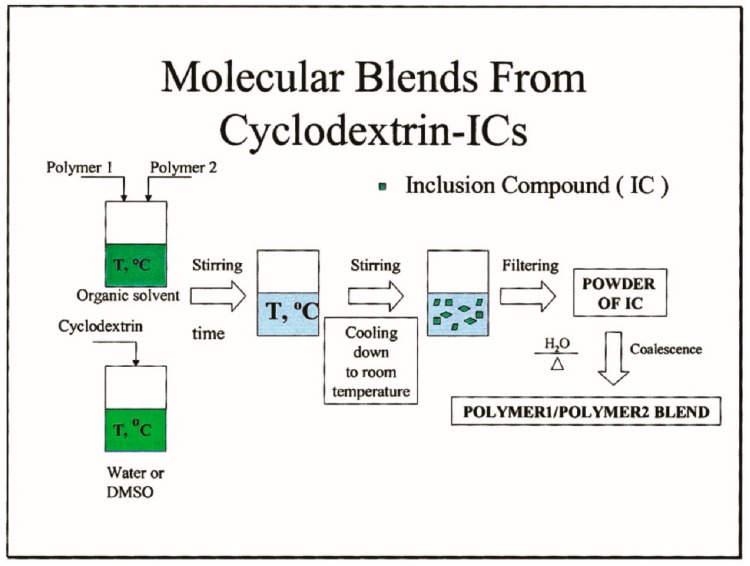
Schematic depiction of the formation of polymer1/polymer2 blends via coalescence from their common CD–ICs. Reproduced with permission from [28]. Copyright 2004 Wiley Periodicals, Inc.

As indicated in Figure 8, when two or more polymers are dissolved in the same solvent and are gradually combined with a solution of CD or U, usually an aqueous or methanol solution, we may form CD- or U-IC crystals that include and contain both or more polymers [23,28]. When the crystalline CD or U lattice is carefully removed from the common ICs, the included polymers are initially coalesced into well-mixed blends. 

This procedure has been applied to achieve a variety of well-mixed polymer blends, including poly(l-lactic acid) (PLLA)/PCL [29], polycarbonate (PC)/poly-styrene (PS) or PMMA [30], atactic-poly(β-hydroxy butyrate) (PHB)/PCL [31], poly (ethylene terephthalate) (PET)/poly(ethylene 2,6-naphthalate) (PEN) [32], PC/PMMA [33], PVAc/PMMA/PC [33], PVAc/PMMA [23,34] or PC [34], and nylon-6/nylon-6,6 [35]. 

All of these well-mixed coalesced blends were formed with inherently incompatible polymers that could not be solution or melt blended without phase separation of their component polymers, and yet were resistant to phase separation caused by high temperature annealing above ther T_g_s and T_m_s [28]. The remaining discussion of well-mixed blends obtained by coalescence from common CD–ICs will be restricted to the PLLA/PCL binary blend [28,29,36], whose behaviors are representative of all those listed above. 

**Figure 9:**
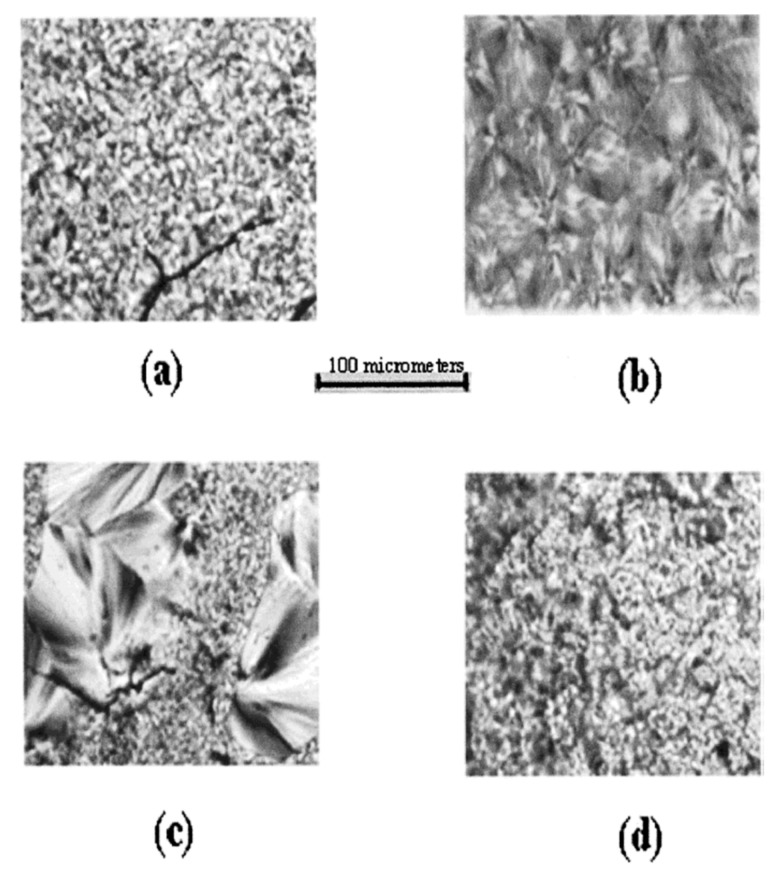
Polarizing photomicrographs of melt pressed (**a**) poly(l-lactic acid) (PLLA), (**b**) PCL, (**c**) solution-cast, and (**d**) melt pressed coalesced PLLA/PCL blends. Reproduced with permission from [29]. Copyright 2012 American Chemical Society.

Figure 9 presents polarized micrographs of melt pressed films of PLLA (a) and PCL (b), a solution cast PLLA/PCL film (c), and a melt- pressed film of coalesced PLLA/PCL (d). Their comparison indicates a lack of and substantial mixing of PLLA and PCL, respectively, in the solution-cast and melt-pressed coalesced PLLA/PCL films.

**Figure 10:**
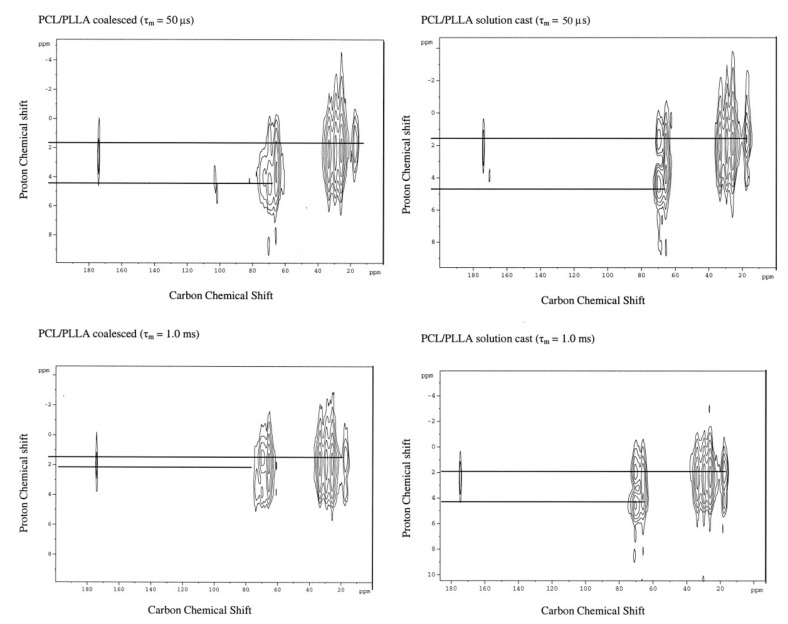
2D ^1^H-^13^C heteronuclear correlation (HETCOR) spectra of solution-cast and. Coalesced PCL/PLLA blends. Reproduced with permission from [28]. Copyright 2004 Wiley Periodicals, Inc.

Figure 10 presents the 2D-^1^H-^13^C heteronuclear correlation (HETCOR) NMR spectra of the solution-cast and coalesced PCL/PLLA blends observed with short (τ_m_ = 50 μs) and long (τ_m_ = 1.0 ms) mixing times [37,38,39]. A normal ^1^H–^13^C correlation spectrum is observed with the shorter mixing time for both coalesced and solution-cast blends, where the PLLA methyl and PCL methylene proton chemical shifts are clearly distinguished. In the HETCOR spectrum observed with the longer mixing time for the coalesced PCL/PLLA blend, however, these two proton chemical shifts approach each other. This is a clear indication of effective spin-diffusion between them, which requires spatial proximity [36,37,38,39]. 

Even when a long mixing time is employed, the HETCOR spectrum of the solution-cast blend does not show significant proton spin diffusion between PCL and PLLA chains. The coalesced and solution cast PCL/PLLA blends, respectively, are clearly intimately mixed and phase separated, as revealed by efficient proton spin diffusion in the former blend and the absence of spin diffusion in the latter blend.

The intimacy of mixing in the largely amorphous PCL/PLLA blends produced by coalescence from their α-CD–IC crystals and those cast from their common solution were estimated [36]. This was achieved through use of a two-dimensional HETCOR spin-diffusion technique [40]. Rates of intrapolymer polarization transfer vs. interchain/interdomain polarization equilibration were readily differentiated using this two dimensional (2D) heteronuclear NMR technique. This enabled spin-diffusion coefficients and the length scales of miscibility to be determined by direct measurement.

The resulting length scales of mixing in the coalesced and solution cast PCL/PLLA blends were 4.9 and 7.4 nm, respectively. Radii of gyration for the PCL and PLLA chains investigated were expected to be 3.5–5.0 nm, which are consistent with the length scale of mixing in the coalesced blend. In contrast, the length scale of mixing in the solution cast PCL/PllA blend (7.5 nm) exceeds the radii of gyration of both polymers, so clearly only the PCL and PLLA chains in the coalesced blend are molecularly mixed.

PCL and PLLA are biocompatible and biodegradable, but individually, neither possess favorable mechanical performance. PCL has a low T_m_ and a low tensile strength, while PLLA has a much higher T_m_, but it has low ductility and is brittle. Furthermore, they are inherently incompatible with each other, as seen in their solution cast blend (Figure 9c. However, in the PCL/PLLA blend coalesced from its common α-CD–IC, the component chains are intimately mixed and remain so even after heating well above their T_m_s at 200 °C for 12 h [29]. 

**Figure 11:**
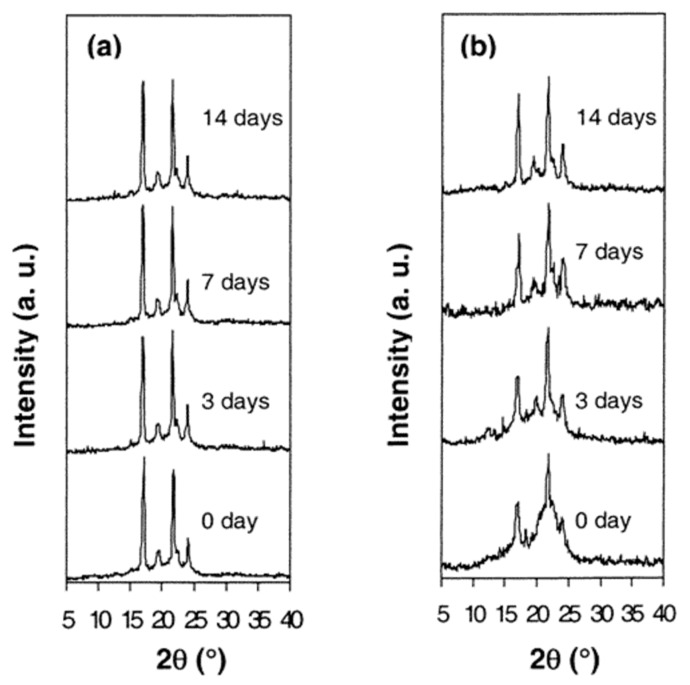
X-Ray diffraction patterns of as-synthesized (**a**) and coalesced (**b**) PCL-b-LLA films, after various enzymatic degradation times. Reproduced with permission from [41]. Copyright 2003 American Chemical Society.

A PCL-b-PLLA di-block copolymer was synthesized and its α-CD–IC was formed [41]. Upon coalescence of the di-block copolymer, the PCL and PLLA blocks were observed to be substantially mixed rendering the bulk coalesced sample largely amorphous, as seen in the wide angle X-ray diffractograms of as-synthesized and coalesced PCL-b-PLLA in Figure 11. 

Both di-block samples were treated with a *lipase* enzyme that only degrades the amorphous sample regions, and in Figure 11 we can see before enzyme treatment that the coalesced sample was largely amorphous. Upon treatment with the enzyme the amorphous regions were removed, so after two weeks of treatment the initially largely amorphous coalesced sample now gave a diffractogram that appears semicrystalline. This example illustrates the ability of the formation of and coalescence from a block copolymer-IC can control its biodegradation. 

The hope that such well-mixed polymer blends made by coalescence from their common ICs may not be a complete “pipe dream” may in fact be realized. Fibers melt-spun from PCL coalesced from its U-ICs showed significantly improved mechanical properties [42]. Because of the much higher weight fraction of guest polymer in their U-ICs, compared with their CD–ICs, and the ready availability of U, coalescence of bulk samples from polymer-U-ICs may be practical.

Like the PCL/PLLA blend [29], each of the polymer blends that were formed by coalescence from their common CD- and U-ICs [30,31,32,33,34,35] were also found to be well-mixed and thermally stable. We suggested previously that the extremely sluggish center-of-mass diffusion that must accompany full entanglement of the coalesced chains may be the source of the subsequent slow establishment of homogeneous well-mixed melts [21,26]. Apparently the process of entangling the largely separated and not fully interpenetrating randomly coiled chains coalesced from their CD–ICs is in fact much slower than the center-of mass diffusion of polymer chains in their fully entangled melts. 

Each of the well-mixed blends obtained by coalescence from their common CD-and U-ICs were also stable to high temperature annealing, even though they were formed from inherently immiscible polymers. Even the additional thermodynamic driving force experienced by the initially coalesced and well-mixed polymer chains is not sufficient to rapidly reorganize them into their thermodynamically favored phase separated melts. 

### 3.2. Single Polymer Composites

**Figure 12:**
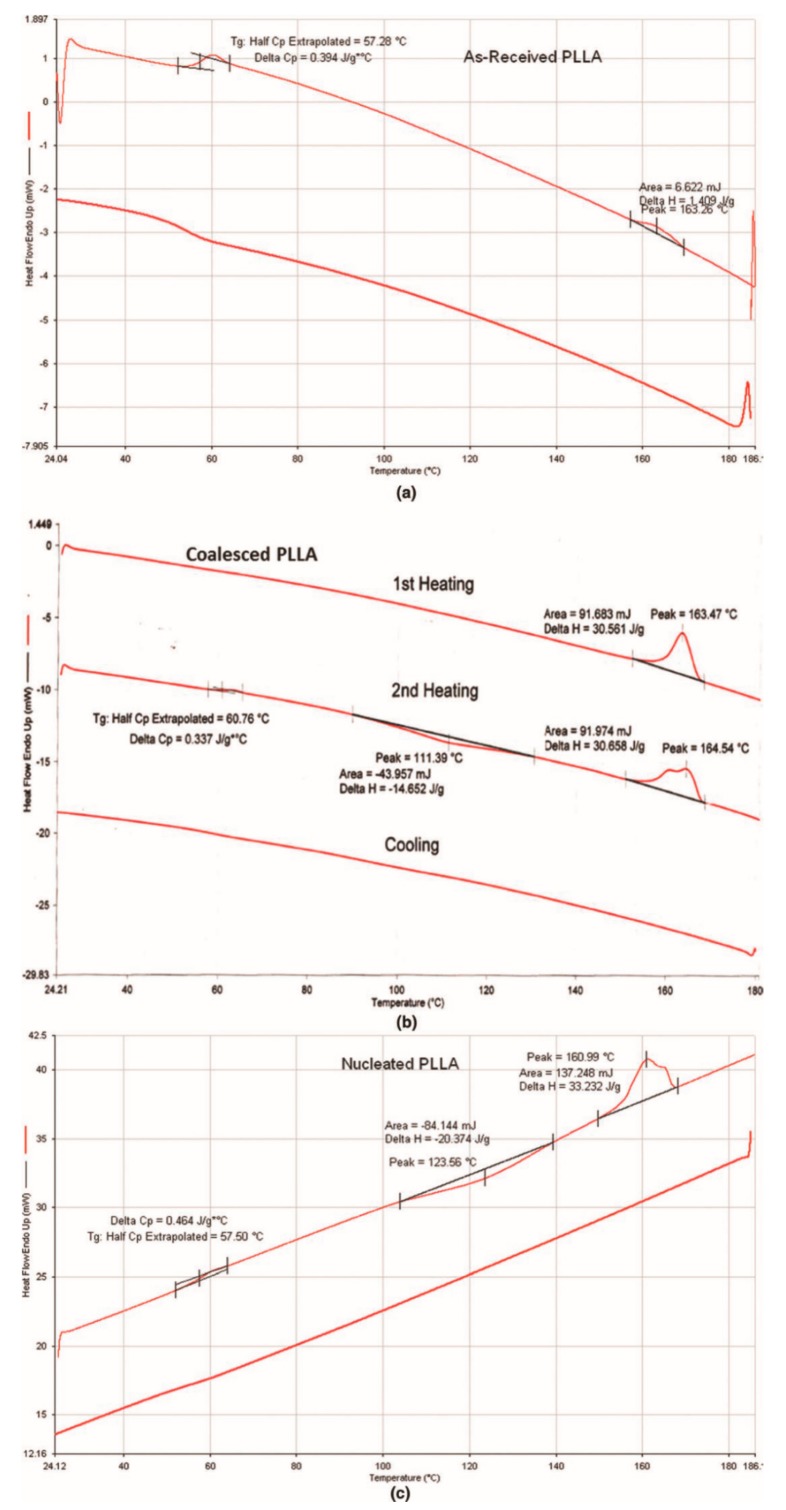
First cooling and 2nd heating DSC scans (10 °C/min) of asr-, c-, and nuc-PLLA samples from top to bottom. Nuc-PLLA contains 2 and 98 wt% of c-PLLA and asr-PLLA. Reproduced with permission from [27]. Copyright 2012 Wiley Periodicals, Inc.

Semi-crystalline polymers coalesced from their crystalline ICs can be used as self-nucleants to enhance the melt crystallizability of as-received (asr) samples. Figure 12, and previously Figure 6, illustrate this, where in Figure 12 the 1st cooling and 2nd heating scans (10 °C/min) of PLLA are presented. While the asr-PLLA is nearly completely amorphous, c- and nuc-PLLAs are able to crystallize from the melt upon cooling and cold crystallize further in the solid above T_g_.

Single polymer composite sandwich samples consisting of one layer of asr-polymer film and one layer of nuc-polymer film were constructed. The tensile results shown for single layer PCL films are presented in Table 1 [4], while for two-layer PCL film sandwiches the results can be seen in Table 2. Neat c-PCL and nuc-PCL films have higher moduli than single asr-PCL films and reduced elongations at break. Table 2 shows that asr-PCL/c-PCL and asr-PCL/nuc-PCL film sandwiches are stronger and less extensible than the asr-PCL/asr-PCL control film sandwich. 

No delamination was observed in the tensile tests. In addition, T-Peel tests were performed on the PCL sandwiches, and all three evidenced virtually equal film interface strengths.

**Figure 13:**
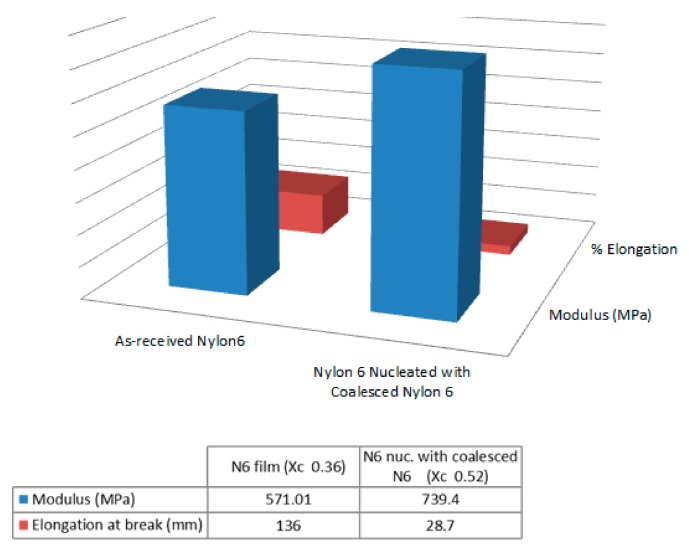
Moduli and elongation at break of asr- and nuc-nylon-6 films, where the latter film contained 2 wt% c-Nylon-6 and 98 wt% asr-nylon-6 obtained with permission from [44,45]. Copyright 2010, Elsevier Ltd and 2011, American Chemical Society.

**Figure 14:**
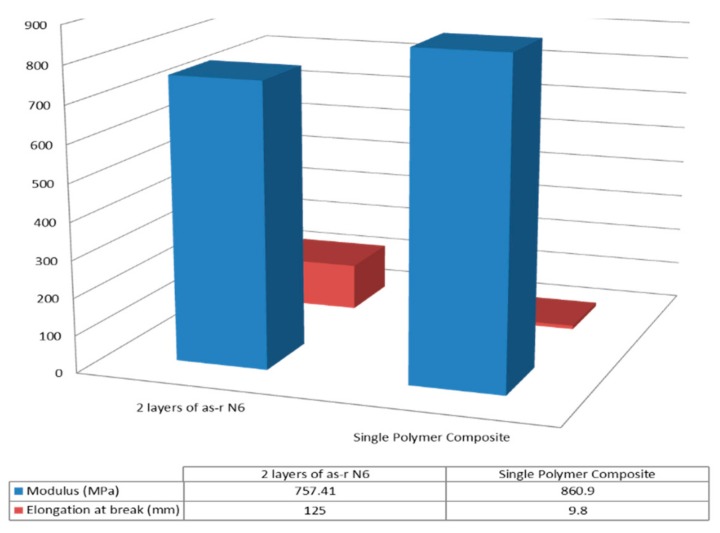
Moduli and elongation at break of melt pressed asr/asr and asr/nucl Nylon-6 film sandwiches. Reproduced with permission from [44]. Copyright 2005 American Chemical Society.

**Figure 15:**
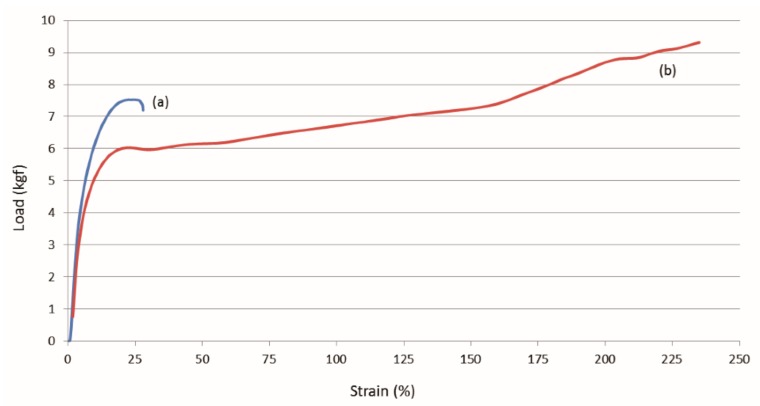
Strain responses of asr-N-6/nuc-N-6 (**a**) and asr-N-6/asr-N-6 (**b**) film sandwiches to applied loads obtained with permission from [45]. Copyright 2011 American Chemical Society.

Single polymer nylon-6 (N-6) film composites were similarly fabricated and their mechanical behaviors observed [44,45]. Moduli and elongation at break of the 60,000 MW N-6 films are presented in Figure 13, where the nuc-N-6 film containing 2 wt% c-N-6 is seen to be stronger and less extensible than the asr-N-6 film. A similar result is seen in both Figure 14 and Figure 15 for the asr/asr and asr/nuc-N-6 film sandwiches. 

**Figure 16:**
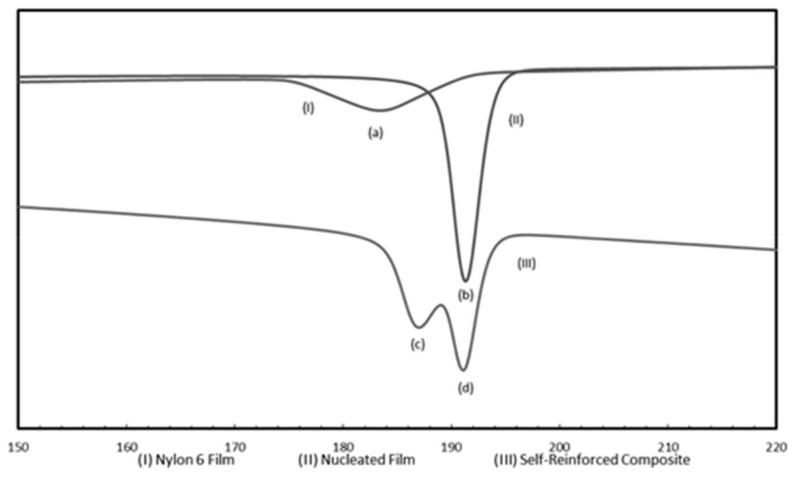
DSC cooling scans from the melts of (I) asr-N-6, (II) nuc-N-6, and (III) asr-N-6/nuc-N-6 sandwich films. Peaks (**a**), (**b**), (**c**), and (**d**) correspond to *T*_c_s of 183.5, 191.5, 186, and 191.1 °C, respectively. Reproduced with permission from [45]. Copyright 2011 Elsevier Ltd.

Finally in Figure 16 DSC cooling scans from the melts of asr-N-6 and nuc-N-6 films and the two layer asr-N-6/nuc-N-6 film sandwich are presented [45]. Each sample spent a total of 10 min in the melt before cooling at 10 °C/min, and provide clear evidence that the asr/nuc-N-6 sample sandwich does not become homogeneously mixed during melt processing. Clearly nuc-polymers can serve as reinforcement for asr-matrices of the same polymer.

Polymer-polymer composites were also formed by embedding polymer A-CD- or U-ICs into films and fibers made from a second polymer B, followed by solvent removal of the host CD or U. The embedded films were made by melt processing at temperatures above the T_m_ of polymer B and below the decomposition temperature of the polymer A-IC. A solvent for the CD or U hosts that does not dissolve either polymer was used to remove them, yielding a composite of polymer B embedded with polymer A [46,47].

**Figure 17:**
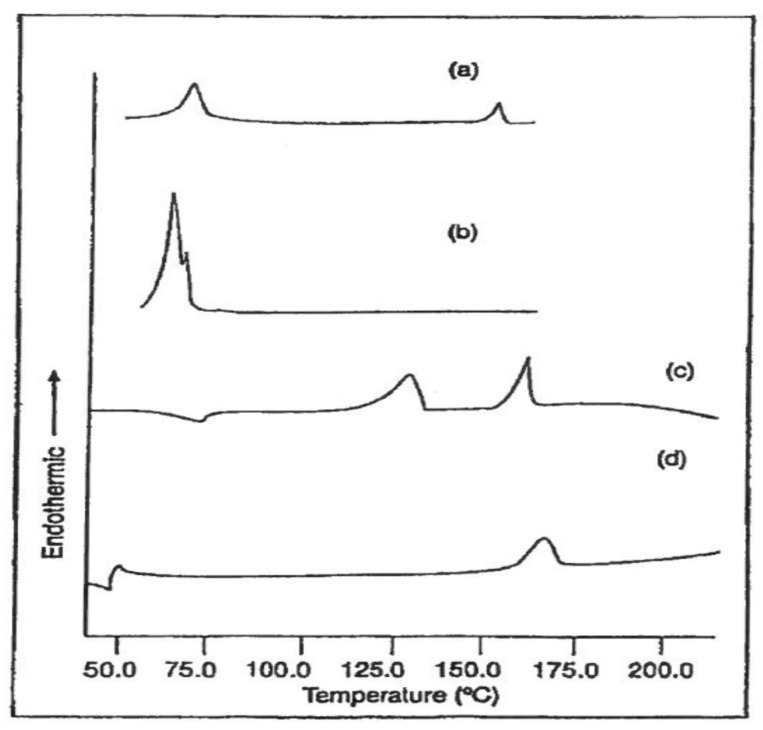
DSC scans of PCL embedded with PCL-U-IC before (**a**) and after (**b**) washing in methanol, and DSC scans of PLLA embedded with PCL-U-IC before (**c**) and after (**d**) washing in methanol obtained with permission from [47]. Copyright 2001 WILEY.

As examples of this approach, in Figure 17 are presented the DSC scans of PCL and PLLA films embedded with PCL-U-IC before and after washing in methanol [47]. The higher and lower temperature endotherms in (a) and (c) are produced by melting of the embedded PCL-U-IC crystals, which are absent in (b) and (d) after soaking in methanol. Only PCL melting is observed after soaking the PCL embedded film (b), and only PLLA melting is evident in the methanol soaked embedded PLLA film. While melting of the coalesced PCL expected after soaking the embedded PLLA film in methanol is not observed in the DSC scan (d), solid-state ^13^C-NMR observations not presented here do confirm the presence of a small amount of coalesced PCL [47].

The water vapor permeabilities of pure and U- or PCL-U-IC embedded PCL and PLLA films observed before and after soaking in methanol can be seen in Table 3. Removal of U from the U embedded PCL and PLLA films leads to large increases in their water vapor permeabilities, presumably through holes created by removing the U crystals. On the other hand, the films embedded with PCL-U-IC do not evidence significant increases in permeabilities after soaking in methanol. These observations suggest that the PCL coalesced from its U-IC crystals upon soaking in methanol fills in or heals any holes created by the removal of the host U. Similar results were observed for PLLA and nylon-6 films embedded with the α-CD–IC of poly(ethylene oxide) before and after washing in hot water [47]. 

These examples make clear that it is possible to obtain polymer composites by embedding a polymer B-CD or U-IC into polymer A and then soaking it in a solvent that only dissolves the IC host. In this manner we can control the compositions and structures of polymer blends to achieve unique morphologies that are substantially different from those possible by the usual solution casting and melt blending approaches. 

## 4. Summary and Conclusions

The use of small host molecules to molecularly restructure polymer materials at the nanometer level were described here. Among such small molecule hosts, we focused on the cyclic starches CDs and U to form non-covalent crystalline ICs with a variety of polymers. 

Guest polymers are separated, highly extended, and reside in narrow channels created by the host CD- and U-IC crystals. After the careful removal of the host crystalline lattice, a bulk sample of the coalesced guest polymer chains is obtained, but with an organization that is distinct from that normally produced from its melt or from its solutions. 

Such coalesced polymer samples have amorphous regions of greater density, likely with less chain entanglement and more chain alignment. After cooling from their melts, coalesced amorphous polymers show glass-transition temperatures (T_g_s) that are elevated above those of samples prepared from their solutions or melts. Coalesced samples of crystallizable polymers, upon cooling from their melts, are able to crystallize more rapidly and much closer to their melting temperatures (T_m_s). 

The observation that unique behaviors of coalesced polymers are unexpectedly resistant to extended annealing above their T_g_s and Tms indicates that their use is practical. This permits us to create polymer materials with unique and improved properties, such as amorphous polymers with elevated Tgs and semi-crystalline polymers with finer more uniform morphologies, leading to polymer materials with improved thermal and mechanical properties. 

Self-nucleated melt crystallization can be achieved through use of small amounts of the same polymer made rapidly crystallizable via coalescence from its CD- or U-ICs. Polymer composites with as-received polymer matrices and self-nucleated reinforcements, with strong interfaces between them, can be formed in this way.

In addition, well-mixed blends between any two or more polymers can be achieved through simultaneous formation and subsequent coalescence from their common ICs. This occurs despite their inherent propensities for not mixing. Such coalesced and initially well-mixed blends are also resistant to phase segregation when heated for extensive periods well above their T_g_s and T_m_s. As a result, materials produced from them have permanently new and improved properties and behaviors.

## Figures and Tables

**Table 1:** Tensile test results for single layer films. Reproduced with permission from [43]. Copyright 2015 American Chemical Society.

Sample	Modulus (MPa)	Elongation at Break (mm)
asr-PCL 80	344.7 ± 34	247.5 ± 38 (495%)
nuc-PCL 80 (2 wt% c-PCL80, 98 wt% asr-PCL 80)	369.7 ± 40	220.6 ± 65 (441%)
asr-PCL70-90	158.6 ± 8	445.5 ± 17 (891%)
neat c-PCL70-90	210.5 ± 11	420.6 ± 36 (841%)

**Table 2:** Tensile test results for bilayer films obtained with permission from [43]. Copyright 2015 American Chemical Society.

Sample	Modulus (MPa)	Elongation at Break (mm)
asr/asr-PCL 80-film	317.5 ± 24	270.3 ± 30 (540%)
asr/nuc-PCL 80-film	340.7 ± 28	253.6 ± 15 (507%)
asr/asr-PCL 70–90	217 ± 24	415 ± 52 (830%)
asr/c-PCL 70–90 from urea	273 ± 31	404 ± 35 (808%)

**Table 3:** Thicknesses and moisture vapor permeabilities of neat and U- or PCL-U-IC embedded PCL and PLLA films before and after soaking in methanol obtained with permission from [47]. Copyright 2001 WILEY.

Sample Name	Average Thickness (mm)	Moisture Vapor Permeability (g/m^2^/24 h)
PCL film	0.022	375
Dipped PCL film	0.010	440
PCL-Urea film	0.139	413
Dipped PCL-urea film	0.313	747
PCL-IC film	0.054	418
Dipped PCL-IG film	0.076	583
PLLA film	0.024	173
Dipped PLLA film	0.041	187
PLLA-urea film	0.180	207
Dipped PLLA-urea film	0.155	540
PLLA-IC film	0.045	183
Dipped PLLA-IC film	0.052	236

## References

[1] Harada A., Kamachi M. (1990). Complex formation between poly (ethylene glycol) and α-cyclodextrin. Macromolecules.

[2] Huang L., Tonelli A.E. (1998). Polymer inclusion compounds. J. Macromol. Sci. Part C Polym. Rev..

[3] Rusa C.C., Rusa M., Peet J., Uyar T., Fox J., Hunt M.A., Wang X., Balik C.M., Tonelli A.E. (2006). The nanothreading of polymers. J. Incl. Phenom. Macrocyc. Chem..

[4] Fetterely L.C. (1964). Non-Stoichiometric Compounds.

[5] Brown J.F., White D.M. (1960). Stereospecific polymerization in thiourea canal complexes. J. Am. Chem. Soc..

[6] White D.M. (1960). Stereospecific polymerization in urea canal complexes. J. Am. Chem..

[7] Farina M. (1961). Polyhydrotriphenylene. Tetrahedron Lett..

[8] Sozzani P., Comotti A., Bracco S., Simonutti R. (2004). Cooperation of multiple CH⋯π interactions to stabilize polymers in aromatic nanochannels as indicated by 2D solid state NMR. Chem. Commun..

[9] Abe A., Bracco S., Comotti A., Corradini P., De Jeu W.H., De Rosa C., Furuya H., Hiejima T., Kobayashi Y., Li L. (2005). Interphases and Mesophases in Polymer Crystallization II.

[10] Allcock H.R., Levin M.L. (1985). Stereocontrolled polymerization of acrylic monomers within a tris (o-phenylenedioxy) cyclotriphosphazene tunnel clathrate. Macromolecules.

[11] Harada A., Li J., Kamachi M. (1994). Double-stranded inclusion complexes of cyclodextrin threaded on poly (ethylene glycol). Nature.

[12] Shin I.D., Huang L., Tonelli A.E. (1999). Double-stranded inclusion complexes of cyclodextrin threaded on poly(ethylene glycol). Macromol. Symp..

[13] Kawaguchi Y., Nishiyama T., Okada M., Kamachi M., Harada A. (2000). Complex formation of poly (ε-caprolactone) with cyclodextrins. Macromolecules.

[14] Harris K.D., Jonsen P. (1989). ^2^H NMR investigation of the dynamic behaviour of *n*-hexadecane in its urea inclusion compound. Chem. Phys. Lett..

[15] Tonelli A.E. (2009). Molecular processing of polymers with cyclodextrins. Adv. Polym. Sci..

[16] Hunt M.A., Rusa C.C., Tonelli A.E., Balik C.M. (2004). Structure and stability of columnar cyclomaltooctaose (α-cyclodextrin) hydrate. Carbohydr. Res..

[17] Hunt M.A., Rusa C.C., Tonelli A.E., Balik C.M. (2005). Structure and stability of columnar cyclomaltooctaose (γ-cyclodextrin) hydrate. Carbohydr. Res..

[18] Lu J., Mirau P.A., Tonelli A.E. (2002). Chain conformations and dynamics of crystalline polymers as observed in their inclusion compounds by solid-state NMR. Prog. Polym. Sci..

[19] Rusa C.C., Wei M., Bullions T.A., Shuai X., Uyar T., Tonelli A.E. (2005). Nanostructuring polymers with cyclodextrins. Polym. Adv. Technol..

[20] Tonelli A.E. (2008). Nanostructuring and functionalizing polymers with cyclodextrin. Polymer.

[21] Tonelli A.E. (2012). Restructuring polymers via nanoconfinement and subsequent release. Beilstein J. Org. Chem..

[22] Tonelli A.E. (2019). Non-stoichiometric polymer-cyclodextrin inclusion compounds: Con-straints placed on un-included chain portions tethered at both ends and their relation to polymer brushes. Polymers.

[23] Joijode A.S., Antony G.J., Tonelli A.E. (2013). Glass-transition temperatures of nano-structured amorphous bulk polymers and their blends. J. Polym. Sci. Part B Polym. Phys..

[24] Uyar T., Rusa C.C., Hunt M.A., Aslan E., Hacaloglu J., Tonelli A.E. (2005). Reorganization and improvement of bulk polymers by processing with their cyclodextrin inclusion compounds. Polymer.

[25] Williamson B.R., Krishnaswamy R., Tonelli A.E. (2011). Physical properties of poly (ɛ-caprolactone) coalesced from its α-cyclodextrin inclusion compound. Polymer.

[26] Tonelli A.E. (2009). Organizational stabilities of bulk neat and well-mixed, blended polymer samples coalesced from their crystalline inclusion compounds formed with cyclodextrins. J. Polym. Sci. Part. B Polym. Phys..

[27] Gurarslan A., Joijode A.S., Tonelli A.E. (2012). Polymers coalesced from their cyclodextrin inclusion complexes: What can they tell us about the morphology of melt-crystallized polymers?. J. Polym. Sci. Part B Polym. Phys..

[28] Rusa C.C., Wei M., Shuai X., Bullions T.A., Wang X., Rusa M., Uyar T., Tonelli A.E. (2004). Molecular mixing of incompatible polymers through formation of and coalescence from their common crystalline cyclodextrin inclusion compounds. J. Polym. Sci. Part B Polym. Phys..

[29] Rusa C.C., Tonelli A.E. (2000). Polymer/polymer inclusion compounds as a novel approach to obtaining a PLLA/PCL intimately compatible blend. Macromolecules.

[30] Wei M., Tonelli A.E. (2001). Complex formation of poly (ε-caprolactone) with cyclodextrins. Macromolecules.

[31] Shuai X., Porbeni F.E., Wei M., Bullions T., Tonelli A.E. (2002). Formation of inclusion complexes of poly (3-hydroxybutyrate)s with cyclodextrins. 1. Immobilization of atactic poly (*R*,*S*-3-hydroxybutyrate) and miscibility enhancement between poly (*R*,*S*-3-hydroxybutyrate) and poly (ε-caprolactone). Macromolecules.

[32] Bullions T.A., Edeki E.M., Porbeni F.E., Wei M., Shuai X., Rusa C.C., Tonelli A.E. (2003). Intimate blend of poly (ethylene terephthalate) and poly (ethylene 2,6-naphthalate) via formation with and coalescence from their common inclusion compound with γ-cyclodextrin. J. Polym. Sci. Part B Polym. Phys..

[33] Rusa C.C., Uyar T., Rusa M., Hunt M.A., Wang X., Tonelli A.E. (2004). An intimate polycarbonate/poly (methyl methacrylate)/poly (vinyl acetate) ternary blend via coalescence from their common inclusion compound with γ-cyclodextrin. J. Polym. Sci. Part B Polym. Phys..

[34] Wei M., Shin I.D., Urban B., Tonelli A.E. (2004). Partial miscibility in a nylon-6/nylon-66 blend coalesced from their common α-cyclodextrin inclusion complex. J. Polym. Sci. Part B Polym. Phys..

[35] Uyar T., Rusa C.C., Wang X., Rusa M., Hacaloglu J., Tonelli A.E. (2005). Intimate blending of binary polymer systems from their common cyclodextrin inclusion compounds. J. Polym. Sci. Part B Polym. Phys..

[36] Jia X., Wang X., Tonelli A.E., White J.L. (2005). Two-dimensional spin diffusion NMR reveals differential mixing in biodegradable polymer blends. Macromolecules.

[37] White J.L., Mirau P.A. (1994). Heteronuclear correlation in solid polymers: identification of hydrogen bond donors and acceptors in miscible polymer blends. Macromolecules.

[38] Burum D.P., Bielecki A. (1991). An improved experiment for heteronuclear-correlation 2D-NMR in solids. J. Magn. Reson..

[39] Caravatti P., Braunschweiler L., Ernst R.R. (1983). Heteronuclear correlation spectroscopy in rotating solids. Chem. Phys. Lett..

[40] Jia X., Wolak J., Wang X., White J.L. (2003). Independent calibration of ^1^H spin-diffusion coefficients in amorphous polymers by intramolecular polarization transfer. Macromolecules.

[41] Shuai X., Porbeni F.E., Wei M., Shin I.D., Tonelli A.E. (2002). Formation of and coalescence from the inclusion complex of a biodegradable block copolymer and r-Cyclodextrin. 2: A novel way to regulate the biodegradation behavior of biodegradable block copolymers. Biomacromolecules.

[42] Gurarslan A., Caydamli Y., Shen J., Tse S., Yetukuri M., Tonelli A.E. (2015). Coalesced poly (ε-caprolactone) fibers are stronger. Biomacromolecules.

[43] Gurarslan A., Shen J., Tonelli A.E. (2013). Single-component poly (ε-caprolactone) composites. Polymer.

[44] Mohan A., Gurarslan A., Joyner X., Child R., Tonelli A.E. (2011). Melt-crystallized nylon-6 nucleated by the constrained chains of its non-stoichiometric cyclodextrin inclusion compounds and the nylon-6 coalesced from them. Polymer.

[45] Gurarslan A., Tonelli A.E. (2011). Single component polymer composites. Macromolecules.

[46] Huang L., Vasanthan N., Tonelli A.E. (1997). Polymer-polymer composites fabricated by the in situ release and coalescence of polymer chains from their inclusion compounds with urea into a carrier polymer phase. J. Appl. Polym. Sci..

[47] Huang L., Gerber M., Taylor H., Lu J., Tapaszi E., Wutkowski M., Hill M., Lewis C., Harvey A., Herndon A. (2001). Creation of novel polymer materials by processing with inclusion compounds. Macromolecular Symposia.

